# Supplementary material for: Recycling of Vps68 depends on retromer and Mvp1/SNX8
Source: Biol Open. 2026 Apr 15;15(4):bio062518. doi: 10.1242/bio.062518 (PMC13133770; doi:10.1242/bio.062518)
Supplement: Supplementary information [file biolopen-15-062518-s1.pdf]

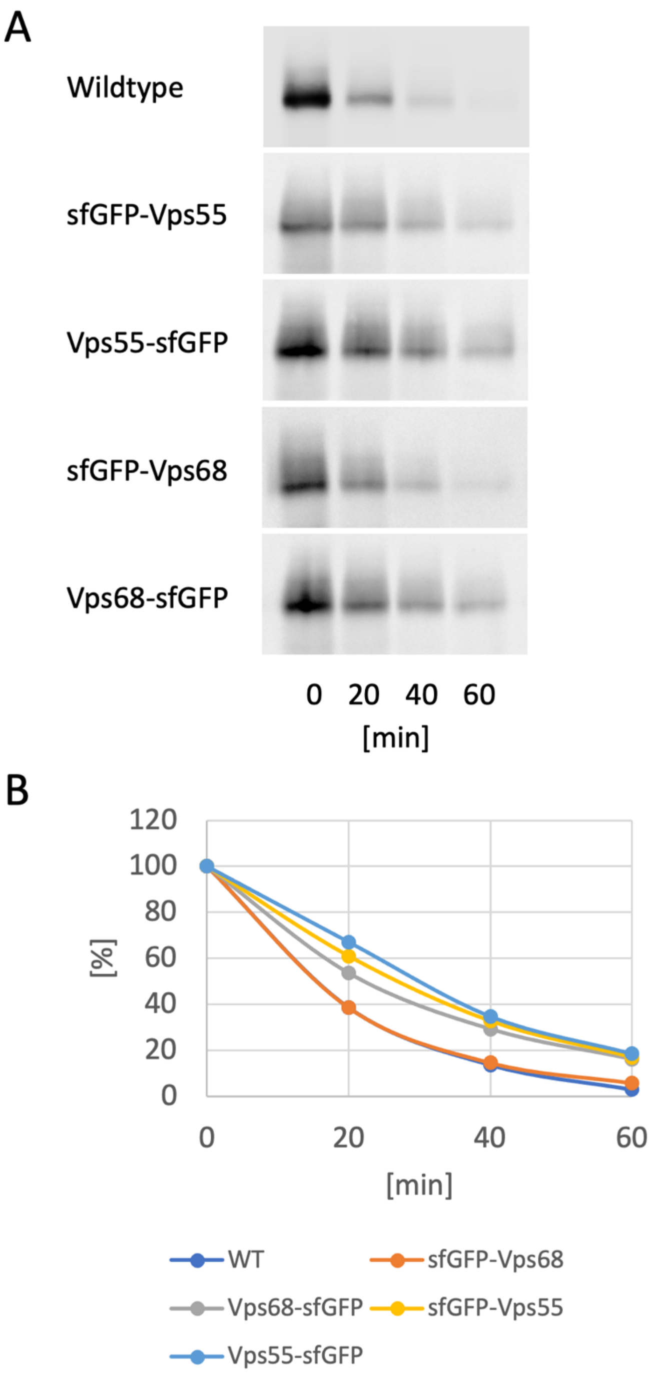

Fig. S1. Influence Vps55 and Vps68 tagging on Ste6 turnover. The Ste6 turnover was examined by a gal-depletion experiment. Cells were first grown in YP-Gal medium and were then shifted to YPD. Cell aliquots were taken at 20 min intervals after a 20-min preincubation in glucose medium and analyzed for Ste6 by western blotting. (A) From top to bottom: RKY3319 (WT), RKY3576 (*sfGFP-VPS55*), RKY3577 (*VPS55-sfGFP*), RKY3574 (*sfGFP-VPS68*), RKY3575 (*VPS68-sfGFP*). (B) Quantification of the western blot signals by ImageJ.

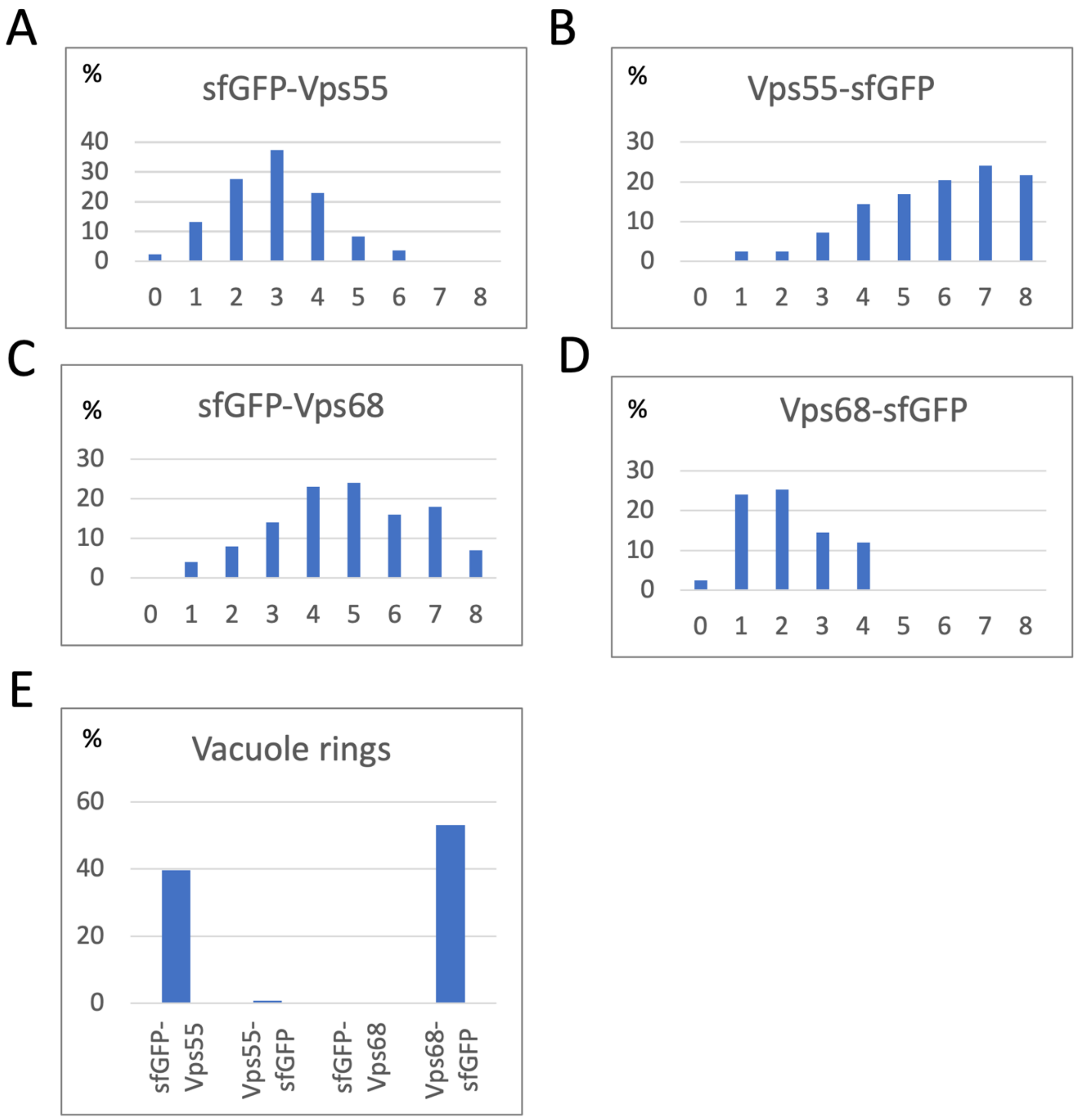

Fig. S2. Number of endosomal dots in cells expressing N- or C-terminally tagged variants of Vps55 and Vps68. The number of fluorescent dots was counted in at least 100 cells for each variant. The percentage of cells with the indicated number of dots is shown. (A) RKY3576 (sfGFP-Vps55), (B) RKY3577 (Vps55-sfGFP), (C) RKY3824 (sfGFP-Vps68), (D) RKY3575 (Vps68-sfGFP), (E) percentage of cells displaying vacuolar rings.

Biology Open • Supplementary information

Table S1. Yeast strains

Strain	Genotype	
BY4741	*MATa his3-Δ1 leu2Δ met15Δ ura3Δ*	EUROSCARF
JD52	*MATa ura3-52 his3-Δ200 leu2-3,112 trp1-Δ63 lys2-801 ssd1-d2*	J. Dohmen, Köln
strains based on BY4741:		
Y06533	*mvp1Δ::kanMX4*	EUROSCARF
Y01386	*snx4Δ::kanMX4*	EUROSCARF
Y01654	*snx3Δ::kanMX4*	EUROSCARF
strains based on JD52:		
RKY2074	*Δvps35::HIS3*	this study
RKY3319	*ste6::[kan GAL1p-STE6]*	this study
RKY3320	*ste6::[HIS3 GAL1p-STE6] Δvps68::kan*	this study
RKY3379	*ste6::[kan GAL1p-STE6] Δvps55::HIS3*	this study
RKY3435	*ste6::[TRP1 GAL1p-STE6] Δvps55::HIS3 Δvps68::kan*	this study
RKY3574	*ste6::[kan GAL1p-STE6] vps68::[HIS3 SNF7p-sfGFP-VPS68]*	this study
RKY3575	*ste6::[kan GAL1p-STE6] VPS68-sfGFP::HIS3*	this study
RKY3576	*ste6::[kan GAL1p-STE6] vps55::[HIS3 SNF7p-sfGFP-VPS55]*	this study
RKY3577	*ste6::[kan GAL1p-STE6] VPS55-sfGFP::HIS3*	this study
RKY3597	*ste6::[kan GAL1p-STE6] VPS55-sfGFP::His3 Δvps35::TRP1*	this study
RKY3795	*ste6::[kan GAL1p-STE6] MVP1-sfGFP::His3*	this study
RKY3824	*vps68::[VPS68p-sfGFP-VPS68]*	this study
RKY3852	*vps68::[VPS68p-sfGFP-VPS68] Δvps35::His3*	this study
RKY3853	*vps68::[VPS68p-sfGFP-VPS68] Δmvp1::His3*	this study
RKY3854	*vps68::[VPS68p-sfGFP-VPS68] Δvps55::His3*	this study
RKY3855	*ste6::[kan GAL1p-STE6] VPS55-sfGFP::His3 Δmvp1::TRP1*	this study
RKY3856	*ste6::[kan GAL1p-STE6] VPS55-sfGFP::His3 Δvps68::TRP1*	this study
RKY3871	*ste6::[kan GAL1p-STE6] VPS55-sfGFP::His3 vps68-ΔCT::TRP1*	this study
RKY3877	*vps68::[VPS68p-sfGFP-vps68-ΔCT::TRP1]*	this study
RKY3895	*VPS55-sfGFP::kan*	this study
RKY3941	*MVP1-sfGFP::kan VPS26-13myc::His3*	this study
RKY3966	*VPS5-sfGFP::kan VPS26-13myc::His3*	this study
RKY3977	*Δsuc2::kan*	this study
RKY3978	*Δsuc2::kan Δvps10::His3*	this study

Biology Open • Supplementary information

Table S2. Plasmids

Plasmid	Vector	Insert
YCplac22		*CEN ARS TRP1*
YCplac33		*CEN ARS URA3*
pRK1691	YCplac22	*sfGFP*
pRK2067	pRK1691	*VPS10*
pRK2070	pRK1691	*VPS10Δ (codons 1428-1495 deleted)*
pRK2074	pRK2070	*VPS68-N-terminus (codons 1-26)*
pRK2075	pRK2070	*VPS68 C-terminus (codons 172-184)*
pRK2076	pRK2070	*VPS55 N-terminus (codons 1-11)*
pRK2077	pRK2070	*VPS55 C-terminus (codons 119-140)*
pRK2083	pRK2070	*VPS55 loop 2 (codons 55-76)*
pRK2084	pRK2070	*VPS68 loop 2 (codons 77-113)*
pRK2085	pRK2070	*VPS68 C-terminus (codons 172-184, Y178,180A)*
pRK2176	YCplac33	*VPS35*
pRK2196	YCplac33	*PRC1-SUC2*
